# An allergenic plant calmodulin from *Artemisia* pollen primes human DCs leads to Th2 polarization

**DOI:** 10.3389/fimmu.2022.996427

**Published:** 2022-09-29

**Authors:** Yue Zhang, Wenzhi Hu, Dongbo Chen, Ming Ding, Tao Wang, Yaojun Wang, Jiaoni Chi, Zhimin Li, Qiang Li, Chengxin Li

**Affiliations:** ^1^ Chinese PLA Medical School, Chinese PLA General Hospital, Beijing, China; ^2^ Department of Dermatology, Air Force Medical Center, PLA, Beijing, China; ^3^ Department of Dermatology, The First Medical Center of Chinese PLA General Hospital, Beijing, China; ^4^ Peking University People’s Hospital, Peking University Hepatology Institute, Beijing Key Laboratory of Hepatitis C and Immunotherapy for Liver Disease, Beijing, China

**Keywords:** allergy (hypersensitive anaphylaxis), *Artemisia* pollen, calmodulin, dendritic cell, Th2

## Abstract

*Artemisia* pollen is the major cause of seasonal allergic respiratory diseases in the northern hemisphere. About 28.57% of *Artemisia* allergic patients’ IgE can recognize ArtCaM, a novel allergenic calmodulin from *Artemisia* identified in this study. These patients exhibited stronger allergic reactions and a longer duration of allergic symptoms. However, the signaling mechanism that triggers these allergic reactions is not fully understood. In this study, we found that extracellular ArtCaM directly induces the maturation of human dendritic cells (DCs), which is attributed to a series of Ca^2+^ relevant cascades, including Ca^2+^/NFAT/CaMKs. ArtCaM alone induces inflammatory response toward Th1, Th17, and Treg. Interestingly, a combination of ArtCaM and anti-ArtCaM IgE led to Th2 polarization. The putative mechanism is that anti-ArtCaM IgE partially blocks the ArtCaM-induced ERK signal, but does not affect Ca^2+^-dependent cascades. The crosstalk between ERK and Ca^2+^ signal primes DCs maturation and Th2 polarization. In summary, ArtCaM related to clinical symptoms when combined with anti-ArtCaM IgE, could be a novel allergen to activate DCs and promote Th2 polarization. Such findings provide mechanistic insights into Th2 polarization in allergic sensitization and pave the way for novel preventive and therapeutic strategies for efficient management of such pollen allergic disease.

## Introduction

About 20-30% of respiratory allergic diseases are induced by inhaling pollen from anemophilous plants. These allergens may lead to rhinitis, conjunctivitis, urticaria, asthma, and even allergic shock (1), which represent a serious public health problems worldwide (2). Moreover, in recent decades, global warming, air pollution, and urbanization have led to the earlier advent of blooming and pollination periods, and thus further aggravating the occurrence of allergic diseases (3). Hence, identifying new allergens and studying their underlying allergenic mechanisms are of great social and public health significance.

*Artemisia* species are the major causes of seasonal allergic respiratory diseases in the northern hemisphere (4), producing high pollen grain quantities that may travel thousands of miles under certain conditions (5). In northern China, nearly 58% of pollinosis patients are sensitized to four major *Artemisia* species, namely, *A. sieversiana*, *A. annua L.*, *A. lavandulifolia* and *A. vulgaris* (6). Studies have identified that seven families of known allergens in *Artemisia* species are involved in IgE-mediated Type I hypersensitivity (7), including the pectate lyase family, defensin-like family, Ole e 1-like family, non-specific lipid transfer protein 1 family, the pan-allergens profilin, polcalcins and galactose oxidase (8, 9).

Calmodulin is an abundant Ca^2+^ receptor that regulates a multitude of physiological processes. It is a ubiquitous, intracellular calcium-receptive protein that that exists in all eukaryotic cells with high evolutionary conservation among plants and animals (10). In plants, calmodulin is involved in pollen tube growth and orientation (11). The extracellular calmodulin is also considered as a messenger for intercellular signal transduction during pollen germination *via* a heterotrimeric G protein, phosphoinositide, and cytosolic Ca^2+^ pathway (12–14). In animals, extracellular calmodulin is present in body fluids, saliva, urine, and milk (15) and is involved in nerve regeneration (16), vascular activity (17), embryonic development (18), and so on. Interestingly, a recent study has identified allergenic calmodulin in *ash* pollen and *Amaranthus palmeri* pollen (19, 20), suggesting the potential role of calmodulin families as allergens.

In this study, we identified a novel allergenic *Artemisia* calmodulin (ArtCaM) recognized by serum IgE from *Artemisia* allergic patients. Clinically, these ArtCaM serological IgE positive (ArtCaM+) patients with allergic diseases exhibited more complex clinical manifestations and longer duration of symptoms, suggesting ArtCaM may be involved in the pathogenesis of allergic diseases.

Dendritic cells (DCs), linking innate with adaptive responses, are the predominant immune cells in hypersensitivity diseases (21, 22). Activation of DCs is an important event in the induction phase of hypersensitivity. Ca^2+^-mediated signals transduction pathways have a critical regulatory role in DCs responses to different allergens. Transient intracellular Ca^2+^ spike or oscillating waves trigger downstream calmodulin-dependent calcineurin/nuclear factor of activated T cells (NFAT), or calmodulin-dependent protein kinases (CaMKs) induce immature DCs upregulation of surface costimulatory molecules CD40, CD80, CD86, and MHC class II for the initiation and maintenance of adaptive immune responses (23). In addition, MAPK signaling pathways involved in specific allergens induce the activation of DCs (24, 25). In this study, we primarily investigated the immunomodulatory effects of extracellular plant calmodulin on DCs, and explored the underlying molecular mechanisms and crosstalk of multiple signal pathways.

## Materials and methods

### Patients and sera samples

A total of 77 patients, 52 male and 25 female (aged from 18–58 years old), were recruited from Inner Mongolia provinces of China (40° 45′ N, 111° 13′ E) between July 2019 and January 2021. All patients were clinically diagnosed with allergic rhinitis, with or without conjunctivitis, asthma, or dermatitis, and had *Artemisia* pollen-specific Ige (sIgE) > 0.35 IU/mL (BioLISA Allergy Reagent Kit™, HOB Biotech, China). Moreover, 15 healthy volunteers were recruited as control groups. There were no significant differences in age and sex between the patients and healthy volunteers.

The blood samples were collected from the allergic patients and healthy volunteers for isolating serum and peripheral blood mononuclear cells (PBMCs). Information and clinical history of the patients were recorded, and the severity of symptoms was assessed using the total nasal symptom score (TNSS) and allergic rhinoconjunctivitis quality of life questionnaire (RQLQ) (26). TNSS is a brief questionnaire that evaluates the severity of the main symptoms subjective ratings of nasal symptoms including runny nose, nasal congestion, nasal itching, and sneezing. RQLQ is an evaluation of allergic rhinitis patients’ quality of life, which consists of seven sections and 28 score points.

This study was performed in accordance with the Declaration of Helsinki and approved by the Ethical Committee of Air Force Medical Center, PLA (No. 202255YJ01). The written informed consent document was signed by the participants and volunteers.

### Skin prick test (SPT)

Skin prick tests (SPT) were performed with an allergenic extract from *Artemisia* pollen (Allergopharma, Germany) on the volar surface of the patients’ forearm. SPT is an important method for diagnosing IgE-mediated type I metamorphosis. The skin reaction was recorded after 15 minutes. The diluent solution was the negative control, while 10 mg/ml histamine solution was a positive control. SI (skin index) was calculated as follows: SI = wheal diameter of extract (mm)/wheal diameter of histamine (mm), SI < 0.25 was negative, a positive skin reaction was defined as a mean wheal diameter ≥ 3 mm, meanwhile 0.3 ≤ SI < 0.5: +, 0.5 ≤ SI < 1: ++, 1 ≤ SI < 2: +++, SI ≥ 2: ++++.

### Separation and purification of IgE from volunteers’ and patients’ serum

IgE antibodies were purified from the serum pool of 15 healthy controls and 30 allergic patients, respectively. First, polyvinylpyrrolidone (PVP) 40000 was added to the serum at a final concentration of 3% (W/V), which was stirred at 4°C for four hours using a magnetic stirrer. Next, the PVP-treated serum was centrifugated at 17,000 g for 10 minutes to remove β-lipoprotein and globulin from the pellet. The retained supernatant was immediately replaced with antibody binding buffer using ÄKTA pure FLPC system (GE Healthcare, Sweden) with HiPrep 26/10 Desalting Column (GE Healthcare, Sweden). Then, following a previous protocol, IgG/M/A was removed from the solution prepared in the previous step with Protein A chromatography column (GE Healthcare, Sweden) (27), and the flow-through containing IgE was collected. After purification of the Protein L chromatography column (GE Healthcare, Sweden), purified IgE from the healthy controls (cIgE) and allergic patients (pIgE) was obtained, respectively. The purified IgE was visualized with SDS-PAGE gel staining with Coomassie brilliant blue R-250. In addition, the protein concentration was measured by the BCA method.

### Extracts of pollen from *A. sieversiana* and *A. lavandulifolia*

The pollen of two dominant *Artemisia* species in northern China (*A. sieversiana* and *A. lavandulifolia*) was collected (**Figure S1A**). Next, the pollen was defatted with acetone at 4°C for 8 hours, following 24 hours of extraction with 0.125 M ammonium bicarbonate (W/V = 1:20). After the crude protein lysate was clarified by filtration, the samples were lyophilized for 36 hours and stored at -80°C for further application. Finally, the BCA method was carried out to measure the extracted pollen protein concentration. At the same time, the protein samples were analyzed by SDS-PAGE and visualized by staining with Coomassie brilliant blue R-250 as well (**Figure S1B**).

### Transcriptome and proteome of *A. sieversiana* and *A. lavandulifolia*

RNA and protein samples of *A. sieversiana* and *A. lavandulifolia* were prepared as previously described (28). RNA-seq was obtained on the platform of Illumina NovaSeq 6000. Transcriptome sequencing data were obtained using the SOAP *de novo* assembly program and the short-read trinity assembly program, with annotations generated by alignment to NR, Swiss-Prot, GOG, KEGG and GO databases (NCBI accession: PRJNA834888). Samples peptide mass used Q-EXACTIVE (Thermo Fisher, USA) (**Figure S1C**). Spectra were searched against our *A. sieversiana* and *A.lavandulifolia* transcriptome using the Mascot software, with peptide and fragment mass tolerance set to 20 ppm and 0.6 Da, respectively. Sequence data and spectra were generated for created proteome databases UniProt *Artemisia sieversiana* [205378] and UniProt *Artemisia lavandulifolia* [637482] (Shanghai Bioprofile Technology Co., Ltd., China).

### pIgE and pollen antigens Co-IP

We performed pIgE/antigens Co-IP using Capturem™ IP & Co-IP Kit (Takara Bio, USA). Briefly, 100 µg pollen extraction (from *A. sieversiana* and *A.lavandulifolia*) and 100 µg pIgE mixture 500 µL, pre-incubated with anti-IgE monoclonal antibody (ab106494, Abcam, USA), were loaded onto the equilibrated spin column and centrifuged at 1000×g for 1 min at RT. After being washed by wash buffer in kits, the spin column was inserted into the collection tube containing 5 µL neutralization buffers. The eluted sample was collected by using a 50 µL Elution Buffer.

### Liquid chromatography-mass spectrometry (LC-MS) analysis

Six immunoprecipitated antigens samples from *A. sieversiana* (n=3) and *A. lavandulifolia* (n=3) were trypsin digested and then desalted on C18 Cartridges (Empore™ SPE Cartridges, Sigma-Aldrich, USA), and then concentrated by vacuum centrifugation and reconstituted in 10 µL of 0.1% (v/v) formic acid (HPLC Grade, Sigma-Aldrich, USA). MS experiments were performed on a Q Exactive HF mass spectrometer coupled to Easy nLC (Thermo Scientific, USA). The MS data were analyzed using MaxQuant software version 1.6.0.16. MS data were searched against the UniProt *Artemisia sieversiana* [205378] and UniProt *Artemisia lavandulifolia* [637482]. This part of the work was supported by Shanghai Bioprofile Technology Company Ltd., China.

### IgE immunoblot analysis of ArtCaM

ArtCaM with 6×His tag at the N-terminus (**Table S2**) was recombinantly expressed in BL21 (DE3) *E. coli* and directly purified from soluble fraction on Ni-chelating Sepharose chromatography using AKTA purifier (GE Healthcare, Sweden), and the purity was assessed by SDS-PAGE (**Figure S2A**). The LPS level was detected at a concentration of < 10 EU/mg (Genscript Toxinsensor kit, L00350, USA).

The molecular weights of recombinant ArtCaM were identified by Western blot with an exposed protein marker (TDY Biotech, China). ArtCaM (10 μg) was separated by 4-20% gradient SurePAGE™ Bis-Tris precast Gels (GenScript, USA) and electro-transferred onto a PVDF membrane (Millipore, USA) with a pore size of 0.2 μm. The membrane was completely washed with a blocking solution (solarbio, China) to prevent the non-specific binding of antibodies. The membrane was then incubated with serum samples from the healthy controls or allergic patients overnight at 4°C. After being washed with TBST, the membrane was incubated for 1h at room temperature with an anti-human IgE mouse monoclonal antibody (1:6000 in blocking solutions, Abcam, UK). The signals were detected using Enlight™ Western Blot Detection Reagents (Engreen Biosystem, New Zealand) followed by exposure to X-ray film (Kodak, USA).

### *In vitro* human DCs culture and stimulation

PBMCs were isolated by Ficoll-Hypaque density gradient centrifugation (GE Healthcare, Sweden) from the healthy volunteers and allergic patients, respectively. PBMCs were cultured in RPMI-1640 medium supplemented with 10% fetal bovine serum and 1% penicillin/streptomycin in a humidified atmosphere containing 5% CO_2_/95% air at 37°C. On day 1, the non-adherent cells were discarded, and adherent cells were then washed once with PBS and stimulated with GM-CSF (100 ng/ml) and IL-4 (100 ng/ml) to produce immature DCs. On day 5, immature human DCs were induced in the presence of various concentrations (50-200 nM) of ArtCaM, or pIgE (0.5 ug/ml), or LPS (2 ug/ml) as the positive control. After stimulation, DCs and supernatants were collected.

DCs and CD4+ T cells co-culture: The CD14^+^ monocytes and CD3^+^CD4^+^ T cells were sorted from PBMCs by FACS Aria II (BD Biosciences, USA) (**Figure S7**). Subsequently, mixed immature human DCs and CD4+ T cells at rate of 1:10.

### Single-cell calcium fluxes detection

Because human DCs are semi-adherent cells and difficult to visualize the Ca^2+^ fluxes in real-time, we utilized a murine dendritic cell line (DC2.4) to detect the Ca^2+^ fluxes in single-cell resolution. Briefly, DC 2.4 cells were loaded with 5 μM Fluo 4-AM in hanks’ balanced salt solution (HBSS) at 37 °C for 20 min. After washed three times with HEPES, the cells were incubated with HBSS (with 1% FBS) for 40 min. Next, 200 nM ArtCaM was added to the cells. Fluorescence intensity was measured by a laser microscope (Olympus, Japan) at wavelengths of 494 nm (excitation) and 516 nm (emission), and by FACS Aria II Flow Cytometer (BD Biosciences, USA).

### Flow cytometry (FCM)

The DCs were obtained by centrifugation at 350×g for 5 min. The cells were subsequently incubated with PE-conjugated CD80, APC-conjugated HLA, FITC-conjugated CD86, FITC-conjugated CD14, PE-conjugated CD11C, APC-conjugated CD209 and PE-conjugated CD40 DCs stained with monoclonal antibodies at 4°C for 30 min. Finally, the DCs were washed twice, then analyzed by FCM (BD Biosciences, USA). The antibodies are listed in **Table S1**.

### Western blot analysis

After being challenged with ArtCaM/pIgE/IP3R inhibitor (2-Aminoethyl diphenylborinate, 2-APB) for 20 min, the DCs were lysed with RIPA containing a cocktail of proteinase and phosphatase inhibitors. The proteins were separated by 4-20% gradient SurePAGE™ Bis-Tris precast Gels (GenScript, USA) and electro-transferred onto a PVDF membrane (Millipore, USA) with a pore size of 0.2 μm. Immunoreactive proteins were detected by incubating blots with specific Abs. Densitometry analysis of immunoblots was carried out by using Quantity One software (Bio-Rad, USA). The relative levels of pErk1/2, pNFATC2 and pCaMKII were expressed as the ratio to GAPDH, an internal control. The antibodies are listed in **Table S1**.

### Enzyme-linked immunosorbent assay (ELISA)

The DCs treated with 200nM ArtCaM 5 minutes were collected and lysed to detect the levels of IP3 by ELISA kits (Solarbio, China). In addition, the supernatants of DCs culture in 200 nM ArtCaM 48 hours were collected to detect levels of IL-5, IL-6, IL-10, IL-12, IL-13, IL-17 and INF-γ by ELISA kits (Solarbio, China) according to the manufacturer’s instructions. All data were analyzed with the method of a microplate reader (Spectra MR, Dynex, Richfifield, MN). The antibodies and kits are listed in **Table S1**.

### Statistical analysis

Data from parameters were evaluated and indicated as mean ± standard deviation (SD) for three reduplicative experiments. Mean of measurement data conforming to a normal distribution was compared with the Student’s 𝑡 test; ranked data were analyzed by Wilcoxon rank-sum test; clinical data were converted to the constituent ratio by the basic principle of statistical inference, and analyzed by the Chi-Square test. Data analysis was performed with SPSS 22.0, and *p* values < 0.05 was considered statistically significant.

## Results

### Patients’ characteristics

A total of 77 *Artemisia* pollen allergic patients and 15 healthy volunteers who resided in the prairie region of Hohhot, Inner Mongolia, China, were included in this research. Most of the patients (96.10%) showed exorbitant serum total IgE (tIgE >100 IU/ml), all patients had *Artemisia*-specific IgE (sIgE >0.35 IU/ml), and 59.74% patients reached extremely high levels (sIgE >50 IU/ml). About 87.01% of the patients had a positive result in the skin prick test performed with *Artemisia* extracts. The most commonly reported symptom among the patients was rhinitis (100%), followed by conjunctivitis (37.66%), dermatosis (including eczema or urticarial, 20.78%), and asthma (12.99%). The details are described in **Table 1**.

**Table 1:** Chinese patients information (n=77), age, gender, allergy symptoms, allergy symptoms, total IgE levels, specific IgE (for A.vulgaris), skin prick testing (SPT), onset time of allergic symptoms and immunoblot results.

PatientID	Age	Gender	Symptoms^a^	tIgE^b^(IU/ml)	sIgE^c^(IU/ml)	SPT^d^	Onset time^e^	IB^f^
1	26	M	R	>500	>200	++	Jul.	–
2	58	F	C,R,D	>500	>200	++++	Oct.	+
3	30	M	R	>500	>200	++++	Jul.	+
4	38	F	C,R	>500	>200	+++	Jul.	+
5	30	M	AS,C,R	>500	>200	++++	Jun.	+
6	29	F	R	>500	>200	++++	AYR	+
7	26	M	C,R	>500	8.25	++	Aug.	–
8	35	F	C,R	203.77	16.05	++++	AYR	+
9	24	M	C,R	149.94	13	++	Aug.	–
10	29	F	R,D	>500	>200	++++	Sep.	+
11	35	F	R	187.31	15.74	+++	Jul.	+
12	27	F	R	210.94	12.73	++	Aug.	–
13	29	M	C,R,D	>500	1.76	++++	Jul.	+
14	34	M	AS,C,R	>500	2.27	++	Jul.	–
15	24	M	C,R	>500	11.94	++	Aug.	–
16	31	M	R	>500	100.12	+++	Aug.	–
17	27	M	C,R	>500	>200	++	Jul.	–
18	34	F	R	>500	>200	++	Sep.	–
19	45	F	R	494.72	>200	++	Sep.	–
20	19	F	R	189.11	62.14	+	Jul.	–
21	30	M	AS,C,R,D	>500	13	+	Jul.	–
22	30	M	R	>500	>200	–	Sep.	–
23	32	F	C,R,D	>500	>200	+++	Aug.	–
24	36	M	R	>500	>200	+++	Jun.	–
25	23	M	R	>500	>200	+++	Sep.	–
26	23	F	R	>500	>200	+	Jun.	–
27	25	F	R	>500	>200	+	Jul.	–
28	29	F	C,R,D	378.06	12.26	+++	Jul.	+
29	28	M	A,S,C,R	>500	>200	+	Aug.	–
30	20	M	R	>500	>200	+++	Jul.	–
31	34	M	R	>500	>200	++	Jun.	–
32	33	M	R	>500	>200	+++	Jul.	+
33	32	M	AS,C,R,D	>500	>200	+++	Jul.	–
34	37	M	AS,C,R,D	>500	25.2	+	Aug.	–
35	30	M	R	433.54	46.69	+	Jul.	–
36	37	M	C,R	>500	>200	+++	Aug.	–
37	39	F	R	>500	>200	++++	Jul.	–
38	34	M	R,D	>500	>200	++++	Aug.	+
39	36	M	C,R	>500	>200	–	Jun.	+
40	42	F	AS,R,D	>500	>200	+	Sep.	–
41	29	M	AS,C,R,D	>500	>200	–	Jul.	+
42	28	M	R	220.3	25.28	+++	Jul.	–
43	36	F	R	>500	>200	+	Aug.	–
44	22	F	R	>500	>200	+++	Jul.	–
45	20	M	C,R	>500	>200	+	AYR	+
46	26	M	R	>500	43.72	+++	AYR	+
47	29	F	R	20.1	0.39	–	AYR	+
48	40	M	R	40.71	0.35	+	Aug.	–
49	24	M	R	>500	0.56	+	Aug.	–
50	28	M	R	>500	>200	–	AYR	+
51	49	F	R	>500	0.63	++	Jul.	–
52	43	F	R	>500	18.02	+	Aug.	–
53	27	M	D,R	>500	0.38	+	Jul.	–
54	32	M	R	>500	0.54	++	Jul.	–
55	32	M	R	>500	62.46	++	Nov.	–
56	18	F	R	>500	1.88	+++	Jul.	–
57	35	F	R	36.64	0.68	+	Aug.	–
58	25	M	R	>500	>200	+++	Aug.	–
59	30	M	AS,C,R,D	>500	>200	+++	Jun.	–
60	28	M	R	>500	1.75	–	Aug.	–
61	34	M	C,R	473.2	0.44	–	Aug.	–
62	29	M	R	>500	>200	++	Aug.	–
63	35	M	R	>500	>200	++	Sep.	–
64	32	M	C,R	>500	0.37	++	Aug.	–
65	31	M	C,R	>500	>200	+	Aug.	–
66	47	M	AS,C,R	>500	>200	++	Aug.	–
67	36	M	C,R	>500	>200	++	Jul.	–
68	32	M	C,R	>500	>200	++++	Jul.	+
69	24	M	R	>500	>200	++++	AYR	+
70	33	M	C,R	>500	21.98	++	Aug.	–
71	22	M	R,D	>500	>200	++++	Aug.	+
72	26	M	C,R	499.5	0.35	++++	Nov.	–
73	29	M	R,D	>500	>200	–	Oct.	–
74	31	F	D,R	>500	0.4	–	Sep.	–
75	56	F	R	>500	37.94	–	Oct.	–
76	29	M	R	>500	24.38	+	Jul.	–
77	29	M	R	>500	>200	+++	Jun.	+

a. Symptoms abbreviations: AS, asthma; C, conjunctivitis; R, rhinitis; D, Dermatosis: Eczema or urticaria.

b. *In vitro* diagnosis of total IgE assay (IU/ml) by Reversed-enzyme allergo-sorbent test (REAST). (BioLISA Allergy Reagent Kit, HOB Biotech, China).

c. Serum specific IgE assays against Artemisia vulgaris (IU/ml) by REAST (BioLISA Allergy Reagent Kit, HOB Biotech, China).

d. Skin prick testing (SPT) with whole Artemisia pollen extract (Allergo™) on the volar surface of the forearm. SI (skin index) = wheal diameter of extract (mm)/wheal diameter of histamine (mm), SI<0.25 is negative; a positive skin reaction was defined as a mean wheal diameter≥3 mm, meanwhile 0.3≤SI<0.5: +, 0.5≤SI<1: ++, 1≤SI<2: +++, SI≥2: ++++.

e. Onset time of patient’s symptoms; AYR: all year round.

f. IB: Immunoblot results, IgE immunoblot results for ArtCaM; **+**: serum IgE was positive to ArtCaM; **-**: negative result.

### Identification of *Artemisia* calmodulin (ArtCaM) as patients’ IgE-recognized antigen

**Figure 1:**
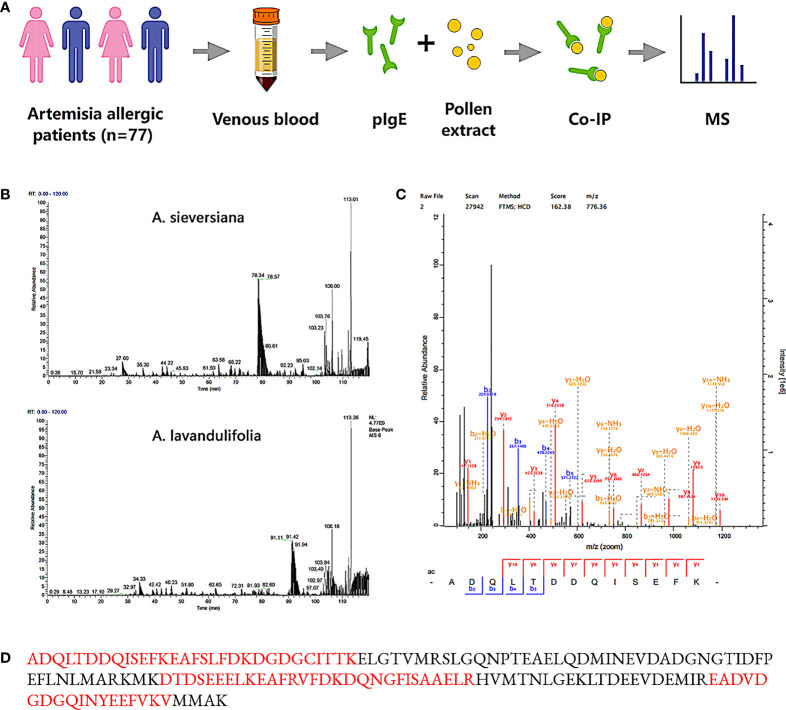
Mass spectrometry and peptide matches. **(A)** Identification process of *Artemisia* calmodulin (ArtCaM). **(B)** MS basepeak of proteins samples from *A.sieversiana* and *A.lavandulifolia* pulled down by patients’ IgE. **(C)** Mass spectra fingerprint of marker peptide “ADQLDDQSEFK”. **(D)** Nature protein sequence, verified peptides by mass spectra fingerprint showed in red.

The purification of IgE from 30 patients whose *Artemisia*-specific IgE (sIgE) was > 50 IU/ml was obtained. Then purified IgE was purified and incubated with the extraction of *A. sieversiana* or *A.lavandulifolia* pollen (n=3 respectively) to capture the potential antigens. Next, IgE-antigen complexes were pulled down with IgE-specific IgG antibody and then analyzed by LC-MS after trypsin digestion (**Figure 1A**). MS identified 28-93 polypeptides from six pollen samples and compared them with the predicted proteome database (UniProt *Artemisia sieversiana/lavandulifolia*, NCBI accession: PRJNA834888). Ultimately, 22 antigens were identified from seven families of known allergens in *Artemisia* species (**Table 2**) and 58 putative antigens.

**Table 2:** Identification of known allergens from *Artemisia* pollen.

Protein names	Protein IDs	Sequence coverage [%]	Sequence length	Score
Non-specific lipid-transfer protein	P0C088	43.5	115	121.64
Putative galactose oxidase	A0A2H4HHX3	19	595	78.904
Profilin-1	Q8H2C9	14.3	133	57.497
Sarcoplasmic calcium-binding protein	P86909	100	8	41.052
Putative galactose oxidase	A0A2H4HI03	11.8	594	40.362
Non-specific lipid-transfer protein	A0A1W5LDC9	43.1	116	38.006
Art v 2.01 allergen	A0A2L1DGY9	18.4	163	29.548
Non-specific lipid-transfer protein	A0A1W5LDB8	28.4	116	27.285
Sarcoplasmic calcium-binding protein	P86909	100	8	22.396
Major pollen allergen Art v 1-like protein	A0A0H3U1U3	13.9	108	13.074
Arginine kinase Lit v 2.0101	Q004B5	3.7	356	10.47
antigen 5 like allergen Cul n 1	A0A6P8KJK7	9.4	255	9.8882
Polcalcin Che a 3	Q84V36	12.8	86	6.6732
Art si 2.0101 allergen	A0A2L1DGX9	6.7	163	6.6515
Major allergen Pru av 1	A0A0B2PZ93	6.3	158	6.4642
Putative Ole e 13.01 allergen	A0A5B6Z6D0	5	484	6.3204
Pectate lyase 3	P28744	2.8	392	6.1919
Minor allergen Alt a 7-like	A0A7J7C8Z2	5.4	203	6.0696
Allergen V5/Tpx-1 family protein	A0A0R2ZEX5	14.8	283	6.0625
Allergen V5/Tpx-1	A0A0K9F466	6.2	373	5.8488
Allergen V5/Tpx-1 related	A0A095AUZ4	6.9	216	5.8317
CRISP/Allergen/PR-1-like Protein	A0A139WGI3	5.1	313	5.8235

A Ca^2+^-binding protein, also named *Artemisia* calmodulin (ArtCaM), repeatedly appeared in all six samples with a score of 162.38 (**Figures 1B–D**). Searching the amino acid sequence of ArtCaM against the NCBI/Protein (https://www.ncbi.nlm.nih.gov/protein/) with BLAST, we found that the antigen was not exclusive in *Artemisia*, but had a universal homology in plants, such as punica granatum, raphanus sativus, prunus persica, vitis vinifera. (**Figure S2B**).

To further research the immunological properties and biologic functions of ArtCaM, we purified ArtCaM in E. coli (**Figure S2A**). The affinity kinetics study showed that ArtCaM was powerfully binding to the patients’ IgE with KD of 4.398×10^-9^ M, while it did not interact with IgE from healthy volunteers (**Figure S2C**).

### ArtCaM-positive patients in IgE Immunoblot had more complex clinical manifestations

**Figure 2:**
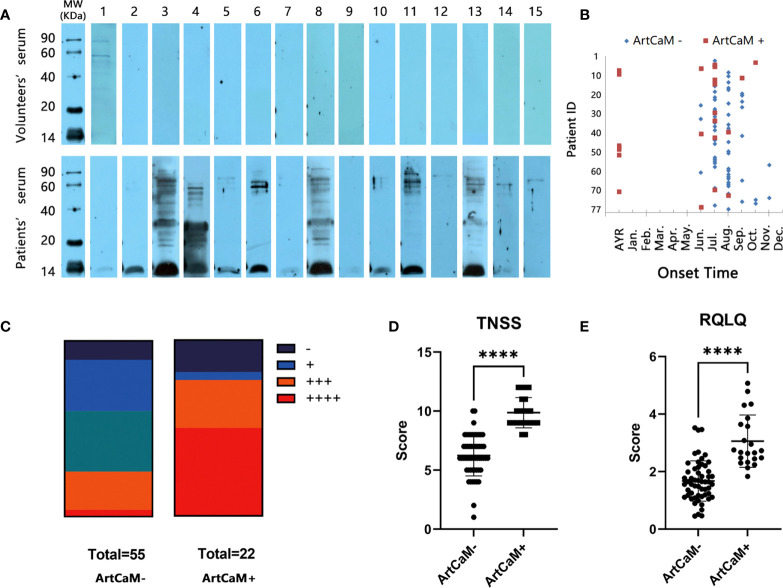
IgE Immunoblot analyses of recombinant ArtCaM. **(A)** Immunoblots of individual serum with *Artemisia* calmodulin (ArtCaM). **(B)** The onset time of ArtCaM- or ArtCaM+ patients’ symptoms, *P<0.0001*. **(C)** Correlation between SPT and Immunoblot results, *P<0.001*. **(D)** ArtCaM-positive patients’TNSS were significant higher than ArtCaM-negative patients. ****P<0.0001. **(E)** RQLQ were significant higher than ArtCaM-negative patients. ****P<0.0001.

ArtCaM was separated by SDS-PAGE and transferred to a PVDF membrane to probe the anti-ArtCaM specific IgE in the serum of *Artemisia*-allergic patients and healthy controls. In positive cases, a characteristically strong signal band was detected around 15 kDa, but was not seen in negative cases (**Figure 2A**). Among 77 individual allergic serum, 22 tests were positive (**Figures 2A**, **S3**, **Table 1**), and all 15 health volunteers’ serum was negative in IgE immunoblot (**Figure 2A**). Statistical analysis showed the anti-ArtCaM IgE was not correlated with the tIgE levels of the patients (both *p>0.1*, **Table S3**, **Table S4**). Furthermore, there was no significant correlation between anti-ArtCaM IgE and specific IgE of other plants (*Artemisia, Sycamore, Elm, Humulus, Ragweed, Cotton, Tobacco, Dandelion, Cypress, Willow*, *p>0.1*, **Table S5**). Among 18 other atopic/allergic individuals with allergic serum, 5 tests were positive (**Figure S6**). However, ArtCaM-positive patients had more complex clinical manifestations. Firstly, ArtCaM-positive patients had a longer duration of symptoms with less seasonality. The symptoms of all ArtCaM-negative patients appeared from June to October, during the flowering phase of local *Artemisia*; but 31.82% of ArtCaM-positive patients had no remission of symptoms throughout the year (*P<0.0001*, **Figure 2B**). Secondly, ArtCaM-positive patients exhibited more severe symptoms. ArtCaM-positive patients had bigger wheal in the skin prick test than healthy controls, implying stronger type I hypersensitivity (*P<0.001*, **Figure 2C**). Moreover, their TNSS and RQLQ scores, which are subjective ratings for severity of nasal symptoms and quality of life, were significantly higher than those of ArtCaM-negative patients (*P*<0.0001, **Figures 2D, E**). Hence, we rationally assumed that ArtCaM not only worked as an allergen but had special immunomodulation effects on allergic reactions.

### ArtCaM induced the maturation of DCs from *Artemisia* allergic patients

There is increasing evidence that DCs mediate an exaggerated Th2 response in allergic airway disease, which contributes to sensitization, initiation and progression of acute allergy and chronic inflammation (29). Airway DCs in the bronchial epithelium capture and present inhaled antigens to naive T cells at mediastinal lymph nodes, which is the basis of the Th2 sensitization process during allergy (30). Therefore, we directly treated DCs with ArtCaM to investigate the biological and adjuvant activity in DCs.

**Figure 3:**
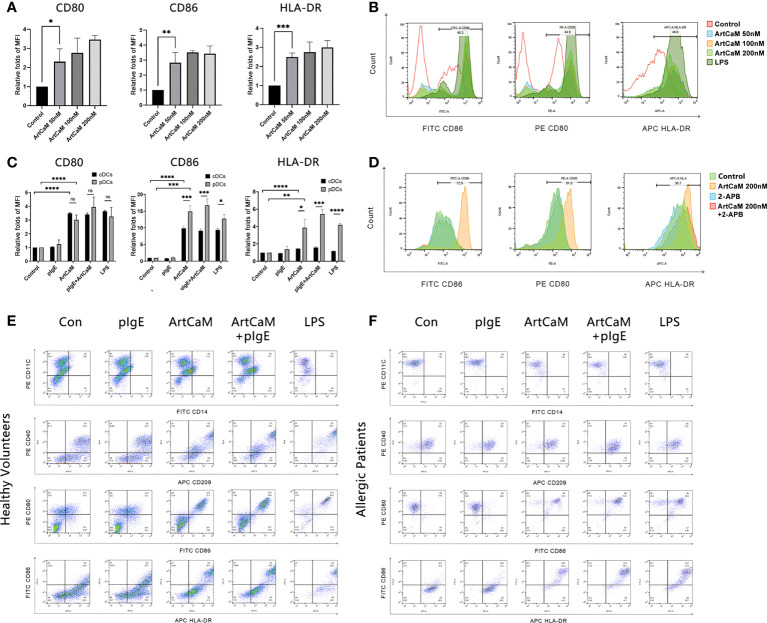
ArtCaM induced DCs phenotypic maturation. **(A)** Statistical diagrams of flow cytometry. The column chart shows the Median Fluorescence Intensity (MFI) by flow cytometryrelative folds of CD80, CD86, and HLA-DR (mean ± SD, n=3) under ArtCaM (0, 50, 100, 200 nM). ns*P*>0.1; *P<0.5; **P<0.01; ***P<0.001; ****P<0.0001. **(B)** Fluorescence intensity changes of CD80, CD86, and HLA-DR under ArtCaM (0, 50, 100, 200 nM) and LPS(2ug/ml). **(C)** Statistical diagrams of flow cytometry. The column chart shows the Median Fluorescence Intensity by flow cytometry relative folds of CD80, CD86, and HLA-DR (mean ± SD, n=3) under ArtCaM, pIgE, ArtCaM+pIgE in both cDCs and pDCs. **(D)** Statistical diagrams of flow cytometry. The column chart shows the fluorescence intensity changes of CD80, CD86, and HLA-DR (mean ± SD, n=3) under control, ArtCaM200nM, 2-APB, ArtCaM200nM+2-APB. 10μM 2-APB significantly reduced the phenotypic maturation of DCs indicated by the CD80, CD86 and HLA-DR levels. **(E)** Phenotype of cDCs under ArtCaM, pIgE, ArtCaM+pIgE, and LPS treatment shows by expression of the antigens (CD40, CD209, CD80, CD86, HLA-DR) on cDCs. **(F)** Phenotype of pDCs under ArtCaM, pIgE, ArtCaM+pIgE, and LPS treatment shows by expression of the antigens (CD40, CD209, CD80, CD86, HLA-DR) on pDCs.

After Human DCs were treated with ArtCaM for 48h, we gated on CD11C+/CD14- cells to distinguish DCs from monocytes and identified DCs using specific markers CD40 and CD209 (31), and 7-Aminoactinomycin D (7-AAD) was also used to distinguish viable cells from dead cells in FCM (**Figure S4A**). Moreover, the maturation of DCs was identified with the expression of class II major histocompatibility complex (HLA-DR) and costimulatory molecules (CD80, CD86) on the surface (32). The maturation of DCs increased in a dose-dependent manner when treated with ArtCaM from 50 to 200 nM (**Figures 3A, B**, **S4B**). We then selected 200 nM ArtCaM as the optimized concentration for the subsequent studies. Although LPS could effectively stimulate the DCs, the distribution of characteristics markers of CD11C, CD14, CD40 and CD209 induced by ArtCaM were obviously different (**Figure S4**), which implied that ArtCaM might be involved in other mechanisms to trigger DCs.

Furthermore, we investigated DCs of healthy controls (cDCs) and *Artemisia* allergic patients (pDCs). In both cDCs and pDCs, ArtCaM significantly increased the levels of costimulatory molecules (CD80 and CD86) and HLA-DR. However, the responses were more sensitive in pDCs (**Figures 3C–F**), which was possibly associated with the patients’ irritable immune microenvironment. Moreover, 200 nM ArtCaM significantly increased the CD80 levels 3.02 folds (*p<0.0001*), CD86 14.90 folds (*p<0.001*), and HLA-DR 3.86 folds (*p<0.01*) in pDCs (**Figure 3C**).

### pIgE did not affect ArtCaM induced DCs maturation

Although pIgE specifically blinds to ArtCaM, it could not affect the ArtCaM-induced maturation of DCs. When pDCs were treated with both ArtCaM and pIgE, the folds of expression of CD80, CD86 and HLA-DR were 3.94, 16.72, and 5.42, respectively. There was no significant difference between ArtCaM and pIgE+ArtCaM groups (*P>0.1*, **Figure 3C**).

### pIgE shifted ArtCaM-induced T cells polarization toward to Th2 responses

**Figure 4:**
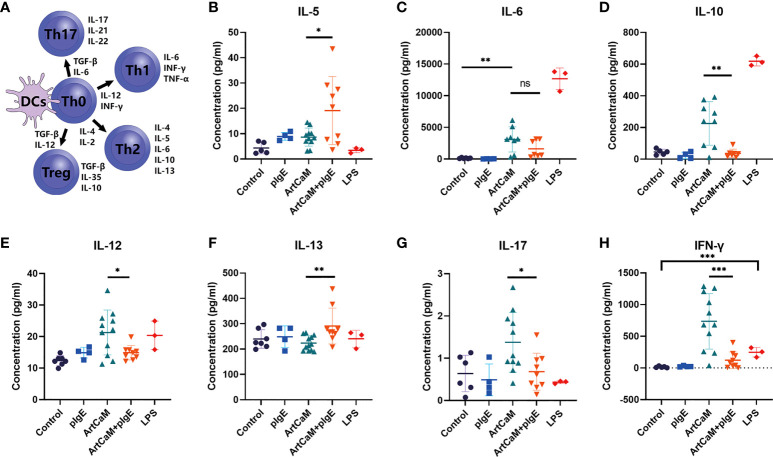
ArtCaM/IgE directed DCs priming Th polarization. **(A)** Schematic diagram of Th polarization. **(B–H)** Cytokines’ concentration secreted by each individual DCs detected by ELISA (mean ± SD, n=3-12). ns*P>*0.1; *P<0.5; ** *P*<0.01; ***P<0.001.

DCs play a crucial role in the priming and differentiation of CD4+ T cells into several distinct subsets, including Th1, Th2, and Th17, as well as regulatory T cells (Treg) (33). Categorization of Th cells is usually based on a dominant cytokine or even a family of cytokines, including Th1 (IL-6, INF-γ, TNF-α), Th2 (IL-4, IL-5, IL-6, IL-10, IL-13), Th17 (IL-17, IL-21, IL-22), and Treg (TGF-β, IL-10, IL-35) (34) (**Figure 4A**). Here, we monitored the dominant cytokine levels to determine the Th polarization. IL-4 was excluded from the research because it was a basic ingredient of medium to culture DCs. After ArtCaM or/and pIgE treatment, Th cells displayed dramatically distinct polarization patterns stimulated by DCs. ArtCaM alone greatly induced secretion of IL-6, IL-10, IL-12, IL-17, and INF-γ, which were associated with Th1, Th17, and Treg polarization, and the patients’ IgE alone had no obvious effects on the secretion of cytokines. However, when ArtCaM was combined with IgE, several well-known Th2 cytokines, such as IL-5 and IL-13, were significantly secreted by DCs, but the Th1/Th17 cytokines were greatly reduced to the baseline of control within 48h (**Figure 4**). The phenomenon indicated that pIgE alone was not sufficient for triggering Th polarization, but could shift the ArtCaM-induced Th polarization from Th1/Th17/Treg to Th2 when combined with ArtCaM.

Hence, although pIgE could not affect ArtCaM-induced DCs maturation, it dramatically redirected the ArtCaM-primed DCs polarization from Th1/Th17/Treg to Th2.

### Putative molecular mechanisms for DCs activation

Calmodulin is a highly conserved protein. The alignment of ArtCaM and human calmodulin showed a similar amino acid sequence with an identity of 89.93%, compared with mouse calmodulin (**Figure S5A**, **Table S2**). Molecule-building of ArtCaM was made through the swiss model (https://swissmodel.expasy.org/) (**Figure S5B**). ArtCaM might cover the similar biological activity of extracellular calmodulin *via* a heterotrimeric G protein, phosphoinositide, and cytosolic Ca^2+^ pathway (12–14). For a preliminary understanding of the molecular mechanisms of ArtCaM-induced DCs activation, we detected several potential target signal pathways of ArtCaM, including Ca^2+^-dependent cascades and relevant ERK pathways (**Figure 6**).

**Figure 5:**
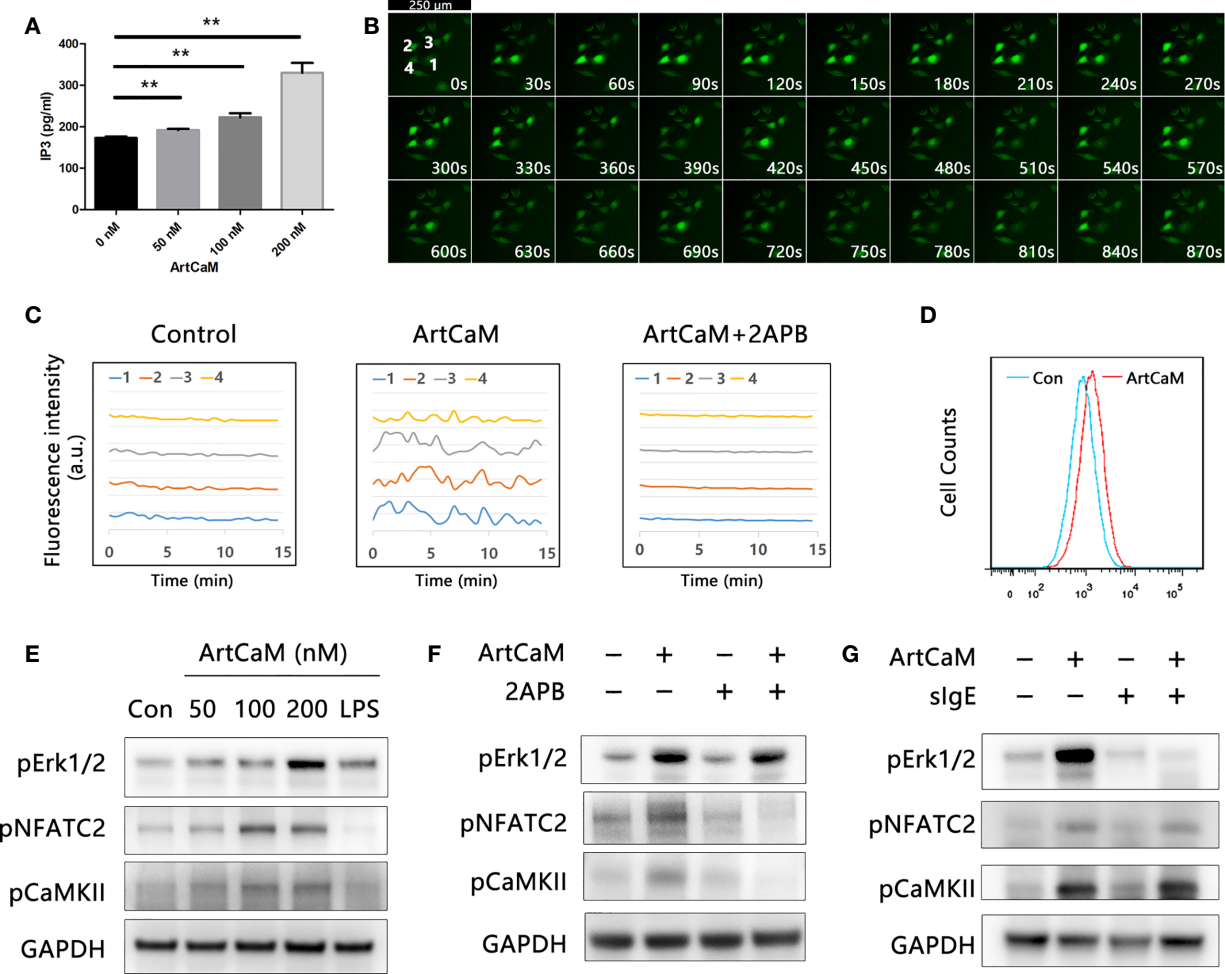
Biological activity of ArtCaM present in DCs. **(A)** ArtCaM in the culture medium was sufficient to increase of intracellular IP3 (**P<0.01). **(B)** Ca^2+^ probe Fluo-4 AM was utilized to monitor the intracellular Ca^2+^ concentration. Fluorescence intensity was measured using laser microscope. Time-lapse in same visual field every 30 seconds which exhibited Ca2+ spikes in individual cells during 15 min. DCs exhibited characteristically oscillatory Ca^2+^ spikes located nearby the nucleus for a period of 2 min. **(C)** Fluorescence intensity changes of four individual cells in control groups, ArtCaM groups, and ArtCaM+2-APB groups.10μM 2-APB significantly reduced the changes of fluorescence intensity. **(D)** ArtCaM induced increasing of Ca^2+^ signal in human peripheral blood DCs detected by flow cytometer. **(E)** ArtCaM induced phosphorylation of Erk1/2, NFATC2, and CaMKII in a dose-dependent manner in minutes. **(F)** IP3R inhibitor 2-APB 10μM significantly reduced the phosphorylation levels of NFATC2, CaMKII, but not Erk1/2 phosphorylation. **(G)** ArtCaM-specific IgE blocked the Erk signal pathway but had no effects on NFATC2 and CaMKII detected by immunoblotting.

Firstly, we detected Ca^2+^-dependent cascades, including inositol 1,4,5-trisphosphate (IP3), intracellular Ca^2+^, and downstream protein kinases. We found that exogenous ArtCaM in the culture medium was sufficient to stimulate the significant increase of intracellular IP3 in 5 minutes, which successively triggered its receptor (IP3R), located on the endoplasmic reticulum (ER), to release Ca^2+^ fluxes (**Figure 5A**, **P<0.01).

Secondly, we utilized the calcium probe Fluo-4 AM to monitor the intracellular Ca^2+^ concentration. ArtCaM-treated DCs exhibited characteristically oscillatory Ca^2+^ spikes near the nucleus for a period of 2 min (**Figures 5B, C**, **S5C, D**). The results were also confirmed in human peripheral blood DCs by FCM, suggesting that ArtCaM instantaneously induced an intense Ca^2+^ signal in minutes (**Figure 5D**). Intracellular Ca^2+^ increase triggered the activity of a series of Ca^2+^-relevant signaling cascades, such as NFAT/CaMKs, which has been proposed to control the immune response and inflammatory process (35, 36). Therefore, we analyzed the phosphorylation levels of NFATc2, CaMKII, and Erk1/2 in human DCs by immunoblot. The results demonstrated that in response to ArtCaM (50-200 nM), the phosphorylation of NFATc2 and CaMKII was significantly increased in a dose-dependent manners, as well as phosphorylation of Erk1/2 (**Figure 5E**).

Thirdly, further research confirmed that Ca^2+^-dependent cascades and Erk1/2 signaling pathways were independent of each other. Because IgE of allergic patients recognized the ArtCaM, we wondered whether sIgE could affect the biological activity of ArtCaM. Therefore, we treated the DCs with pIgE, ArtCaM, or pIgE+ArtCaM. Interestingly, pIgE had no effects on Ca^2+^ cascades, but significantly blocked the activation of Erk1/2 (**Figures 5F, G**).

**Figure 6:**
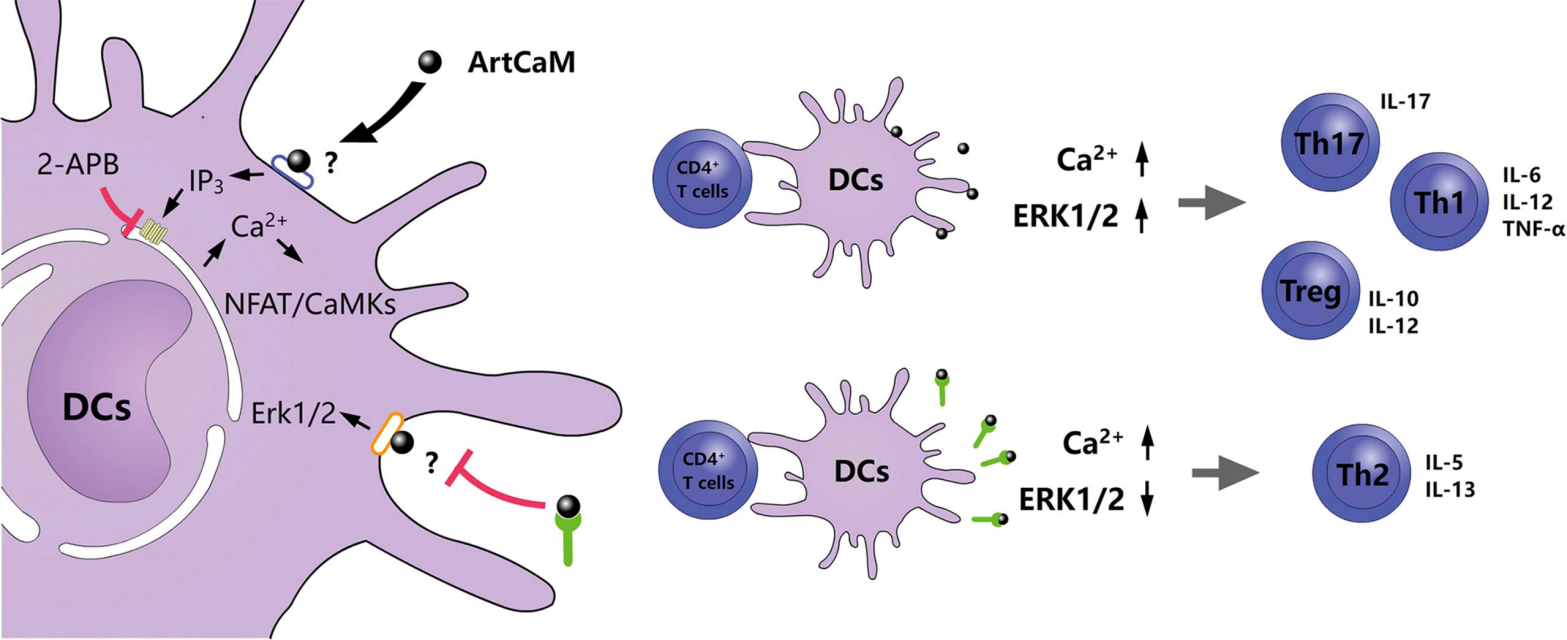
Proposed model of an allergenic plant calmodulin from *Artemisia argyi* pollen primes human DCs leading to Th2 polarization.

Furthermore, when we used a cell-permeable inhibitor of IP3R, 2-APB, at a concentration of 10 μM, the phenotypic maturation of DCs was reduced, which was indicated by the CD80, CD86, and HLA-DR levels *via* flow cytometry (**Figure 3D**). Meanwhile, the Ca^2+^/NFATC2/CaMKII was significantly inhibited, but it had no suppressive effects on Erk1/2. When we utilized 2APB to inhibit the Ca^2+^-dependent cascades, the maturation of DCs was greatly inhibited (**Figure 5F**). Hence, Ca^2+^-dependent cascades were putative signal pathways for DCs maturation; and Erk1/2 pathways were possibly involved in Th polarization (**Figure 6**).

## Discussion

The identification and analysis of new allergens have the potential for immediate application in diagnostic and therapeutic strategies and better understanding of the deep mechanisms of an allergic response and the processes of immune regulation. Recently, the integrated utilization of RNA-sequencing techniques and immune-proteomic approaches have been applied to the raise the identification efficiency for new allergens in several animal and plant species (37). In this study, we used a similar strategy to capture potential antigens by purified IgE from the allergic patients *via* the Co-IP technique. Compared with SDS-PAGE-based immunological identification, this method could exclude the redundant proteins with similar molecular weight to antigens and maintain the natural antigens epitopes denatured by SDS-PAGE produce.

This study identified ArtCaM, a canonical Ca^2+^-binding protein (CBP), accounting for 0.1625 - 0.1858% of whole pollen proteins, by Co-IP and mass spectrometry. CBPs belong to an important protein family, which contains EF-hands motifs to bind and transport Ca^2+^ (38). As a ubiquitous Ca^2+^ sensor, calmodulin is recruited by numerous proteins and enzymes to regulate various cellular events in eukaryotic cells (10). Hence, calmodulin is not exclusive to *Artemisia* but has universal homology in other plants. It has also been identified as a potential allergen in ash and *Amaranthus palmeri* pollen (19, 20). About 28.57% of Artemisia allergic patients’ IgE can recognize ArtCaM, while 27.78% of other allergic patients’ IgE can recognize ArtCaM. IgE from other atopic/allergic individuals have a similar ArtCaM positive rate to *Artemisia* allergy patients (*p>0.1*) Therefore, we assumed ArtCaM might work as a pan-allergen in allergic patients. Meanwhile, CBPs have been identified as highly conserved, immunological and clinical cross-reactivity allergenic proteins in several plants’ pollen (39). Previous research has reported that even though CBPs were only recognized in a few pollen-sensitized patients, they can exhibit stronger allergic reactions and polysensitization in allergic patients (40, 41). This is in line with our findings that more complicated symptoms could be noticed in ArtCaM-positive patients who had stronger hypersensitivity, longer duration, bigger SPT wheal, and higher TNSS/RQLQ scores than in ArtCaM-negative groups. Hence, this ubiquitous eukaryotic extracellular protein calmodulin might be generally involved in the induction phase of allergic sensitization.

Several studies have proved that DCs could orchestrate multiple immune cells including eosinophils, basophils, mast cells and Th cells to initiate the Th2 immune response, in both early-phase allergic reaction and subsequent late-phase allergic reactions (42, 43). DCs present the processed antigens, secret Th2-characteristic cytokines (IL-4, IL-5, and IL-13), and promote class-switching of B cells to produce antigens-specific IgE (44). In this research, we selected the CD11C+ DCs subset, key proinflammatory cells for type 2 inflammation (45), as well as CD40 and CD209, DCs-specific markers which are involved in antigen capture and can promote CD4+ T cells toward Th2 responses (46–48). The physiological concentration of 0.1ug/ul was often used to induce the asthmatic model (49) in mice experiments. We chose 50-200 nM as the optimized concentration for *in vitro* studies. We discovered that biological and adjuvant activity of extracellular ArtCaM contributed to the generation of a pro-inflammatory microenvironment by inducing the maturation of DCs. Furthermore, when ArtCaM combined with pIgE they significantly promoted Th2 polarization.

In this study, the underlying molecular mechanism was further explored. We reasonably presumed that ArtCaM, as a 15 kDa protein, is difficult to enter the cell within a short time. It mostly seems that ArtCaM directly interacts with membrane proteins on DCs to trigger intracellular signal cascades. DCs are key antigen-presenting cells that express a wide variety of membrane proteins, such as pattern-recognition receptors (PRRs), ion channels,and adhesion molecules to adapt extracellular environment and regulate immune function (50, 51). Hence, we identified the putative targets of DCs membrane proteins binding to ArtCaM. The CO-IP between ArtCaM and DCs detected several target proteins with high affinity to ArtCaM, including pattern recognition receptors adaptor protein (WDFY1), ionotropic glutamate receptor (GluR6), and integrins (ITGA2B and ITGB3). These target proteins involved Ca^2+^/IP3 (52) and ERK/MAPK (53) signals which have been confirmed in previous researches. Ca^2+^ concentration plays a critical role in the induction of DCs maturation. We demonstrated that extracellular ArtCaM directly increases the level of secondary messenger IP3, which consequently diffuses into the cytoplasm and binds its receptor IP3R in the endoplasmic reticulum (ER), the largest and most controllable intracellular Ca^2+^ source. The subsequent cytoplasmic localized Ca^2+^ oscillation and dose-dependent activation of Ca^2+^-related signaling pathways, including CaMKs and NFAT, are both crucial for most events in maturation and antigen presentation (36, 54, 55). The cross-validation by IP3R inhibitor 2-APB confirmed that when the IP3R pathway was disturbed, the Ca^2+^ signal and related cascades could be absolutely inhibited. These results suggested that extracellular ArtCaM could directly induce the maturation of dendritic cells (DCs) by a series of Ca^2+^ relevant cascades. Interestingly, we found the ArtCaM triggered the phosphorylation of ERK, but the ERK signaling pathways were independent of Ca^2+^ cascades, since 2-APB did not inhibit the activity of ERK. Furthermore, we found that binding of specific IgE and ArtCaM could block the effects of ArtCaM on Erk-related signals but not Ca^2+^ cascades. The possible mechanism might be that specific IgE blocked the ArtCaM is coincident with the binding site to ERK signal associated membrane proteins (ITGA2B and ITGB3), but not Ca2+ related receptors (WDFY1 and GluR6). On the one hand, ArtCaM as a bioactive protein triggers Ca^2+^-related cascades in DCs. On the other hand, IgE blocks partial signal pathways, such as ERK, making it shift to Th2 polarization to provoke an allergic inflammatory response. However, the exhaustive and precise mechanisms are still unclear and require further validation.

In conclusion, we identified AtrCaM as a putative pan-allergen that could combine with ArtCaM-specific IgE, induce DCs maturation, initiate Th2 polarization, and ultimately exaggerate the allergic responses. Such findings will provide mechanistic insights into Th2 polarization in allergic sensitization, and pave the way for novel preventive and therapeutic strategies for the efficient management of allergic pollen disease.

## Data availability statement

The original contributions presented in the study are publicly available. This data can be found in the NCBI repository, accession number : PRJNA834888. Further inquiries can be directed to the corresponding author.

## Ethics statement

The studies involving human participants were reviewed and approved by the Ethical Committee of Air Force Medical Center, PLA (No. 202255YJ01). The patients/participants provided their written informed consent to participate in this study.

## Author contributions

YZ and WH conceived the study and design the performance of experiments. CL and QL involved in drafting of the article and final approval of the version to be published. YZ and WH collected pollen, performed the gene cloning, natural protein purification and transcriptome/proteome analysis. MD was involved in the clinical study of patients. DC conducted tests and data analyses. JC and ZL collected the recombinant proteins and did the immuno-experiments. TW and YW conducted Cell tests. YZ, WH, and DC drafted the manuscript in close collaboration with all co-authors. All authors contributed to the article and approved the submitted version.

## Funding

This study was supported by a Fund program: Natural Science Foundation of Beijing Municipality, No. 7222186, Beijing, China.

## Acknowledgments

We would like to thank Chengdong Zhang, Shen Zhang, Yu Bai, Yuzhuo Fan and Jiye Hou for technical supports.

## Conflict of interest

The authors declare that the research was conducted in the absence of any commercial or financial relationships that could be construed as a potential conflict of interest.

## Publisher’s note

All claims expressed in this article are solely those of the authors and do not necessarily represent those of their affiliated organizations, or those of the publisher, the editors and the reviewers. Any product that may be evaluated in this article, or claim that may be made by its manufacturer, is not guaranteed or endorsed by the publisher.
