# Supplementary material for: An allergenic plant calmodulin from Artemisia pollen primes human DCs leads to Th2 polarization
Source: Front Immunol. 2022 Sep 29;13:996427. doi: 10.3389/fimmu.2022.996427 (PMC9556433; doi:10.3389/fimmu.2022.996427)
Supplement: Supplementary file 1 [file DataSheet_1.doc]

**Supplementary Materials**

**Table S1.** Antibodies list.

| **Name** | **Brand** | **Cat.** |
| --- | --- | --- |
| Capturem^TM^ IP＆Co-IP Kit | TaKaRa | 635721 |
| Anti-IgE antibody[4C3] | abcam | ab106494 |
| HRP Anti-IgE antibody[1A2] | Abcam | ab106493 |
| Goat Anti-Human IgE H&L (HRP) | Abcam | ab73901 |
| Anti-human IgG H&L（HRP） | Abcam | ab6858 |
| Recombinant Human IL-4 Animal-Free manufactured | PEPROTECH | AF-200-04 |
| Recombinant Human GM-CSF Animal-Free manufactured | PEPROTECH | AF-300-03 |
| BioLISA Allergy Reagent Kit | HOB Biotech | 20210753 |
| HRP conjugated Affinipure Goat Anti-rabbitIgG | BOSTER | BA1055 |
| PE mouse anti-human CD11c | BD Pharmingen | 555392 |
| anti-human CD209 EB-H209 APC | invitrogen by thermo fisher scientific | 17-2099-42 |
| FITC mouse anti-human CD86 | BD Pharmingen | 555657 |
| APC anti-human HLA-DR L243(G46-6) | BD Pharmingen | 559866 |
| PE mouse anti-human CD80 | BD Biosciences | 557227 |
| FITC mouse anti-human CD14 | BD Pharmingen | 555397 |
| anti-human CD40 5C3 PE | eBioscience | 12-0409-42 |
| 7-AAD Staining Solution | BD | 559925 |
| APC anti-human CD4 | BD | 555349 |
| APC anti-human CD3 | BD | 563798 |
| Phospho-CaMKII（T286）（D21E4）Rabbit mAb | Cell Singaling technology | 12716T |
| phospho-NFATC2(ser54) Polyclonal Antibody | invitrogen | 44-944G |
| Rabbit anti-phospho-NFATc2(ser330) | Bioss | Bs-5516R |
| Anti-GAPDH Antibody | BOSTER | A00227-1 |
| Phospho-ERK1/2(Thr202/Tyr204)Polyclonal antibody | proteintech | 28733-1-AP |
| Rabbit Anti-Cyclophilin B Monoclonal Antibody | Bioss | Bsm-52473R |
| 2-Aminoethyl diphenylborinate | MCE | HY-W009724 |
| Fluo-4,AM Calcium Concentration Test kit | Solarbio | CA1190 |
| IP3 ELISA Kit | Solarbio | SEKH-0028 |
| IL-12p70 ELISA kit | Solarbio | SEKH-0021 |
| IL-10 ELISA kit | Solarbio | SEKH-0018 |
| IL-6 ELISA kit | Solarbio | SEKH-0013 |
| IL-5 ELISA kit | Solarbio | SEKH-0012 |
| IL-17A ELISA kit | Solarbio | SEKH-0026 |
| IL-13ELISA kit | Solarbio | SEKH-0022 |
| IL-INFγ ELISA kit | Solarbio | SEKH-0046 |

**Table S2.** Amino acid sequences of Homo sapiens/ Mus musculus /Artemisia calmodulin and recombinant Artemisia calmodulin.

| **Name** | **Amino acid sequences** |
| --- | --- |
| **Calmodulin-3 isoform 1**  **[Homo sapiens]** | MADQLTEEQIAEFKEAFSLFDKDGDGTITTKELGTVMRSLGQNPTEAELQDMINEVDADGNGTIDFPEFLTMMARKMKDTDSEEEIREAFRVFDKDGNGYISAAELRHVMTNLGEKLTDEEVDEMIREADIDGDGQVNYEEFVQMMTAK |
| **Calmodulin-2 isoform 1**  **[Mus musculus]** | MADQLTEEQIAEFKEAFSLFDKDGDGTITTKELGTVMRSLGQNPTEAELQDMINEVDADGNGTIDFPEFLTMMARKMKDTDSEEEIREAFRVFDKDGNGYISAAELRHVMTNLGEKLTDEEVDEMIREADIDGDGQVNYEEFVQMMTAK |
| **Calmodulin**  **[Artemisia]** | MADQLTDDQISEFKEAFSLFDKDGDGCITTKELGTVMRSLGQNPTEAELQDMINEVDADGNGTIDFPEFLNLMARKMKDTDSEEELKEAFRVFDKDQNGFISAAELRHVMTNLGEKLTDEEVDEMIREADVDGDGQINYEEFVKVMMAK |
| **Recombinant calmodulin**  **[Artemisia]** | MGSHHHHHHSGMADQLTDDQISEFKEAFSLFDKDGDGCITTKELGTVMRSLGQNPTEAELQDMINEVDADGNGTIDFPEFLNLMARKMKDTDSEEELKEAFRVFDKDQNGFISAAELRHVMTNLGEKLTDEEVDEMIREADVDGDGQINYEEFVKVMMAK |

**Table S3.** Statistical analysis showed that positive rate of ArtCaM were not correlated with the total IgE levels of patients.

| **tIgE** | **>500** | **<500** | ***P* value** |
| --- | --- | --- | --- |
| cam+ | 18 | 3 | *P>0.1* |
| cam- | 45 | 11 |  |

**Table S4.** Statistical analysis showed that positive rate of ArtCaM were not correlated with the serum specific IgE levels of patients.

| **sIgE** | **1** | **2** | **3** | **4** | **5** | ***P* value** |
| --- | --- | --- | --- | --- | --- | --- |
| cam+ | 1 | 1 | 3 | 1 | 16 | *P>0.1* |
| cam- | 10 | 3 | 5 | 7 | 30 |  |

SIgE 1: 0.35-0.7 IU/mL, SIgE 2: 0.7-3.5 IU/mL, SIgE 3: 3.5-17.5 IU/mL, SIgE 4: 17.5-50 IU/mL, SIgE 5 >50 IU/mL. *P>0.1* as had no significant differences.

**Table S5.** Spearman's nonparametric correlations between *Artemisia* calmodulin (ArtCaM) and other plant allergens.

| **Plants’ name** | | | ***Plant allergens*** | ***Elm*** | ***Scandent hop*** | ***Artemisia argyi*** | ***Ragweed*** | ***Batt*** | ***Tobacco waste*** | ***Dandelion*** | ***Cypress*** | ***Grass pollen mix*** | ***Yang willow*** |
| --- | --- | --- | --- | --- | --- | --- | --- | --- | --- | --- | --- | --- | --- |
| **Spearman's rho** | ***ArtCaM*** | R | -.029 | .129 | -.077 | .153 | .010 | -.112 | .094 | .220 | .030 | -.079 | .063 |
|  |  | *p* | .803 | .264 | .508 | .183 | .928 | .330 | .416 | .155 | .793 | .497 | .583 |

*P>0.1* as had no significant differences.


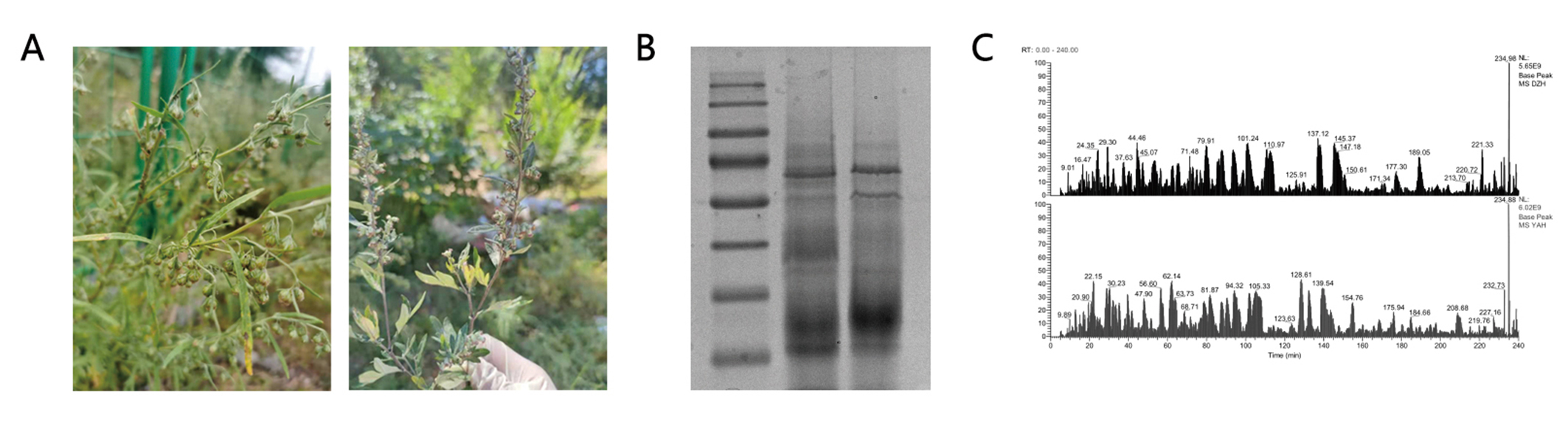


**FIGURE S1** Pollen extracts from *A. sieversiana* and *A.lavandulifolia*. (**A**) Photographs of *A.sieversiana* and *A.lavandulifolia.* (**B**) The protein samples of pollen extracts were analyzed by SDS-PAGE and visualized by staining with Coomassie brilliant blue R-250 as well. **(C**) Liquid chromatography-mass spectrometry (LC-MS) analysis of pollen extracts.


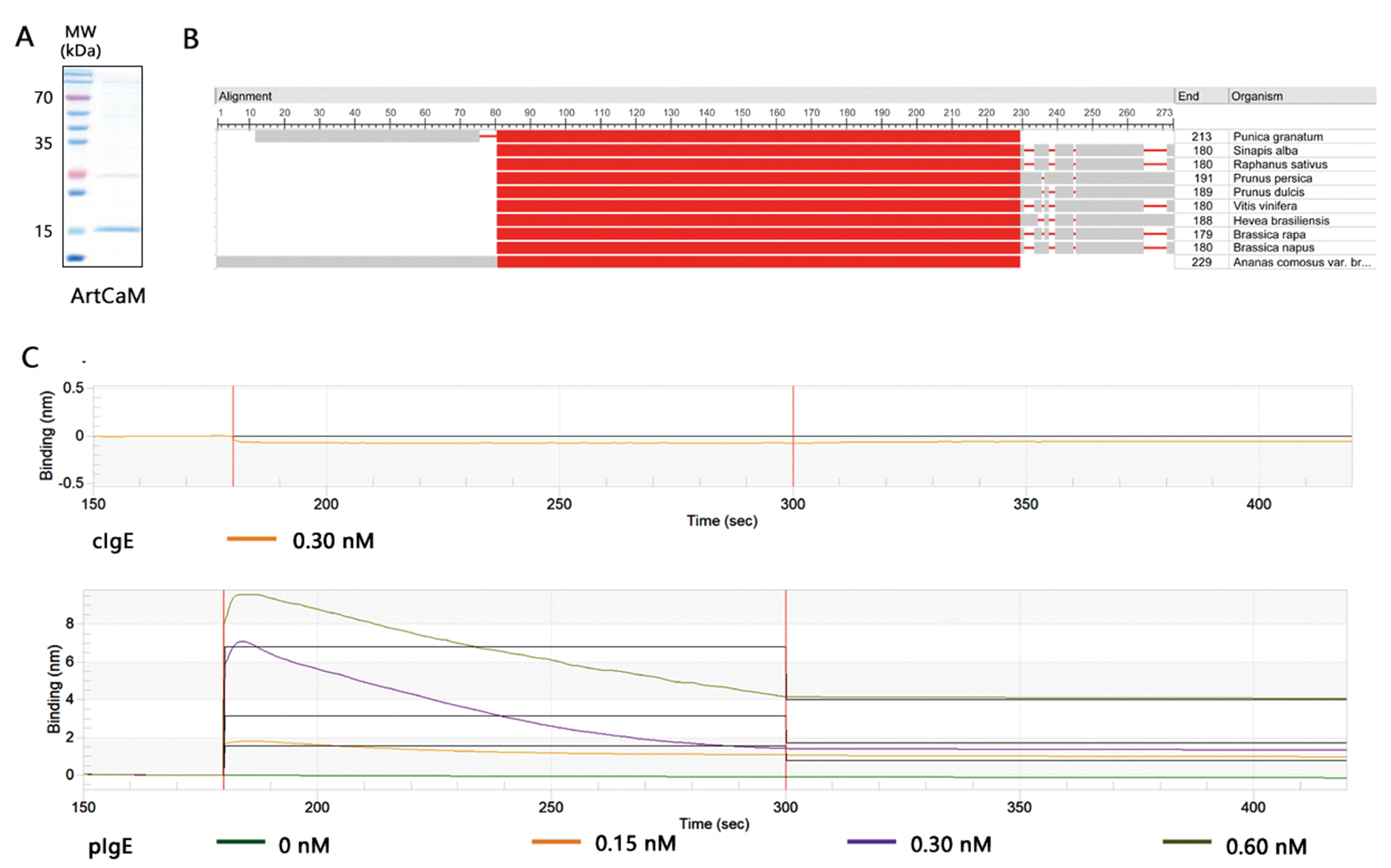


**FIGURE S2** Recombinant ArtCaM. **(A)** Characteristic bands of ArtCaM was analyzed by SDS-PAGE and visualized by staining with Coomassie brilliant blue R-250 at 15KDa. **(B)** Alignment of ArtCaM and plants Calmodulin including punica granatum, raphanus sativus, prunus persica, vitis vinifera. **(C)** The affinity kinetics study of ArtCaM and purified IgE. The KD between ArtCaM patients’ IgE is 4.398×10^-9^ (up), while there is no significant affinity between ArtCaM and healthy volunteers’ IgE.

**
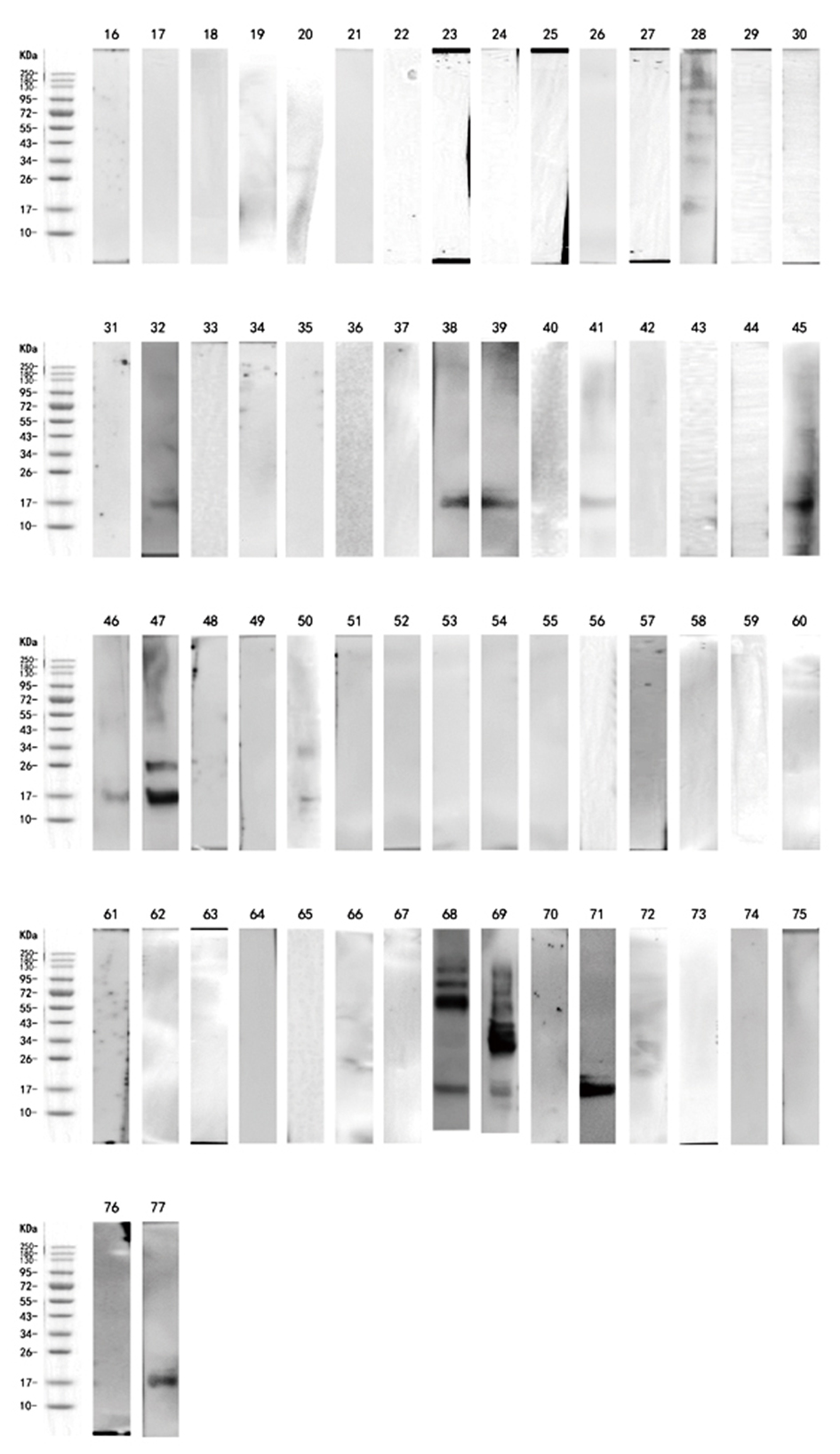
**

**FIGURE S3** Immunoblot probes the ArtCaM-specific IgE in patients’ serum.

**
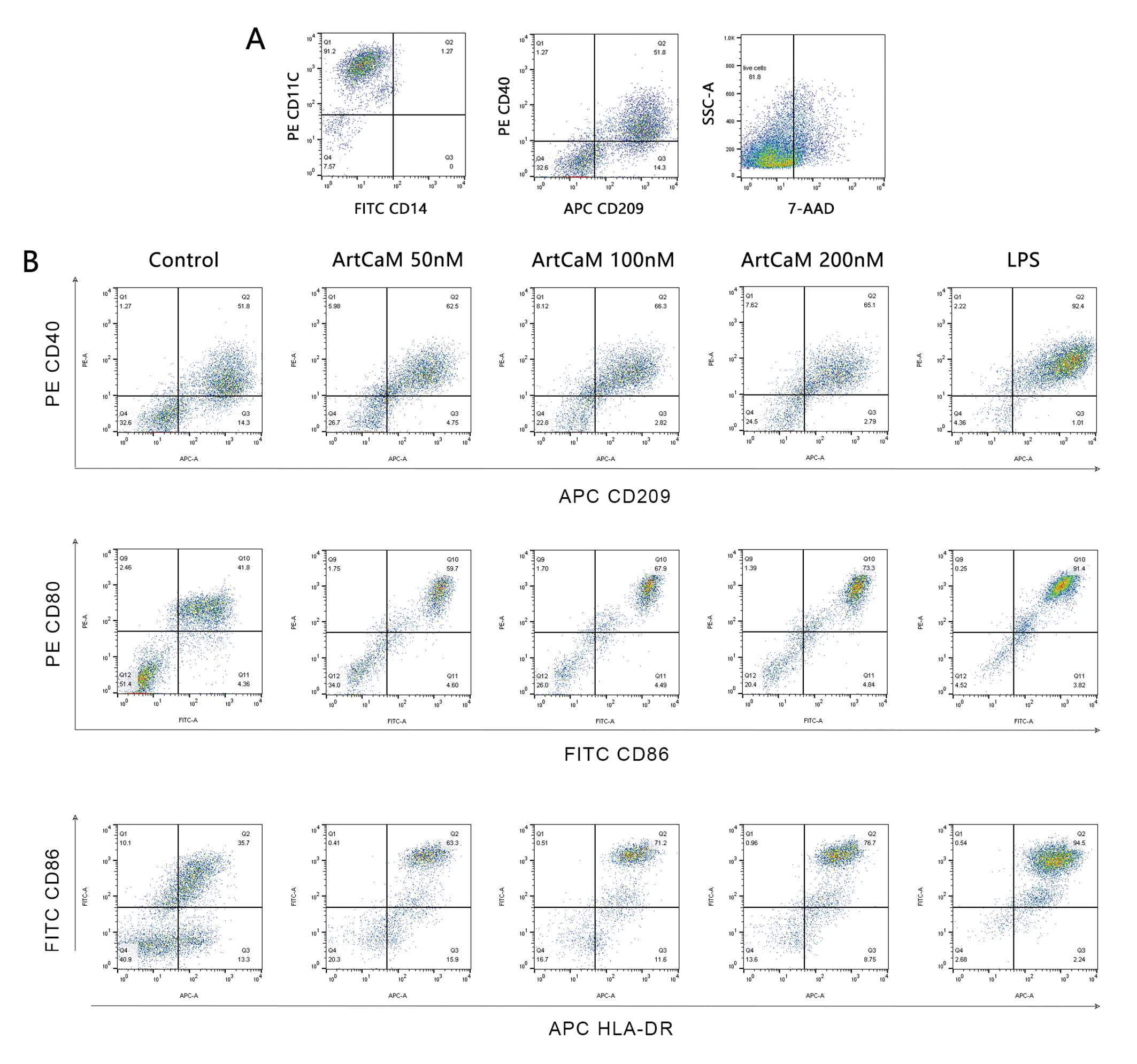
**

**FIGURE S4** ArtCaM induced DCs phenotypic maturation. **(A)** CD11C+/CD14- to mark DCs, 7-Aminoactinomycin D (7-AAD) was used to distinguish viable, apoptotic, and dead cells for in FCM for quality control; **(B)** Phenotype of DCs under ArtCaM (0, 50, 100, 200 nM) and LPS treatment. Dot plots showed the expression of the antigens (CD40, CD209, CD80, CD86, HLA-DR) on DCs.

**
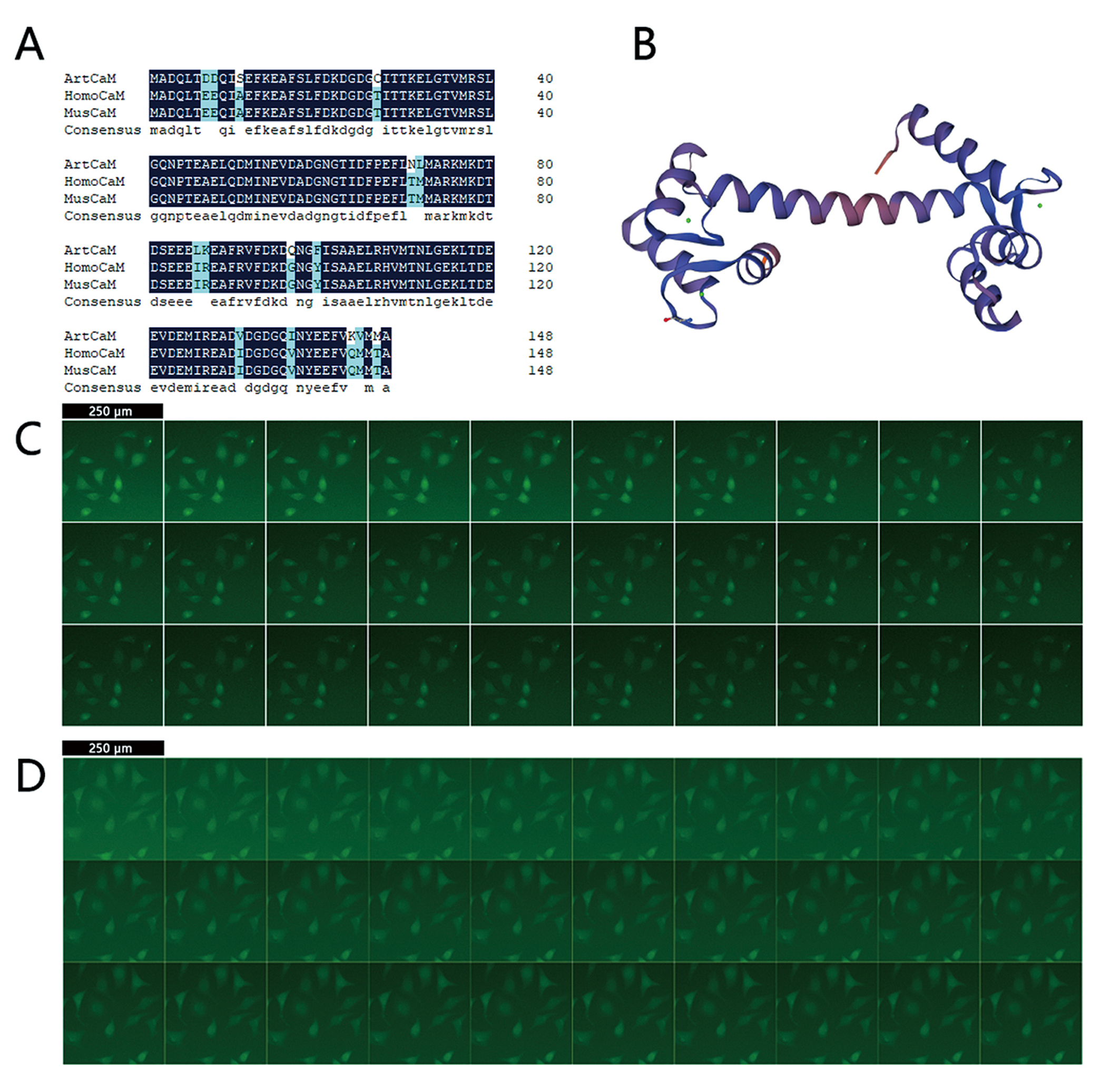
**

**FIGURE S5** Biological activity of ArtCaM present in DCs. **(A)** Calmodulin is a highly conserved protein, the alignment of ArtCaM and human calmodulin showed similar amino acid sequence with identity of 89.93%, as well as mouse calmodulin. **(B)** ArtCaM molecule-building through swiss model (https://swissmodel.expasy.org/). **(C)** Calcium probe Fluo-4 AM to monitor the intracellular Ca^2+^ concentration in control. Fluorescence intensity was measured using laser microscope. **(D)** Calcium probe Fluo-4 AM to monitor the intracellular Ca^2+^ concentration in ArtCaM+2-APB treated DCs. Fluorescence intensity was measured using laser microscope.


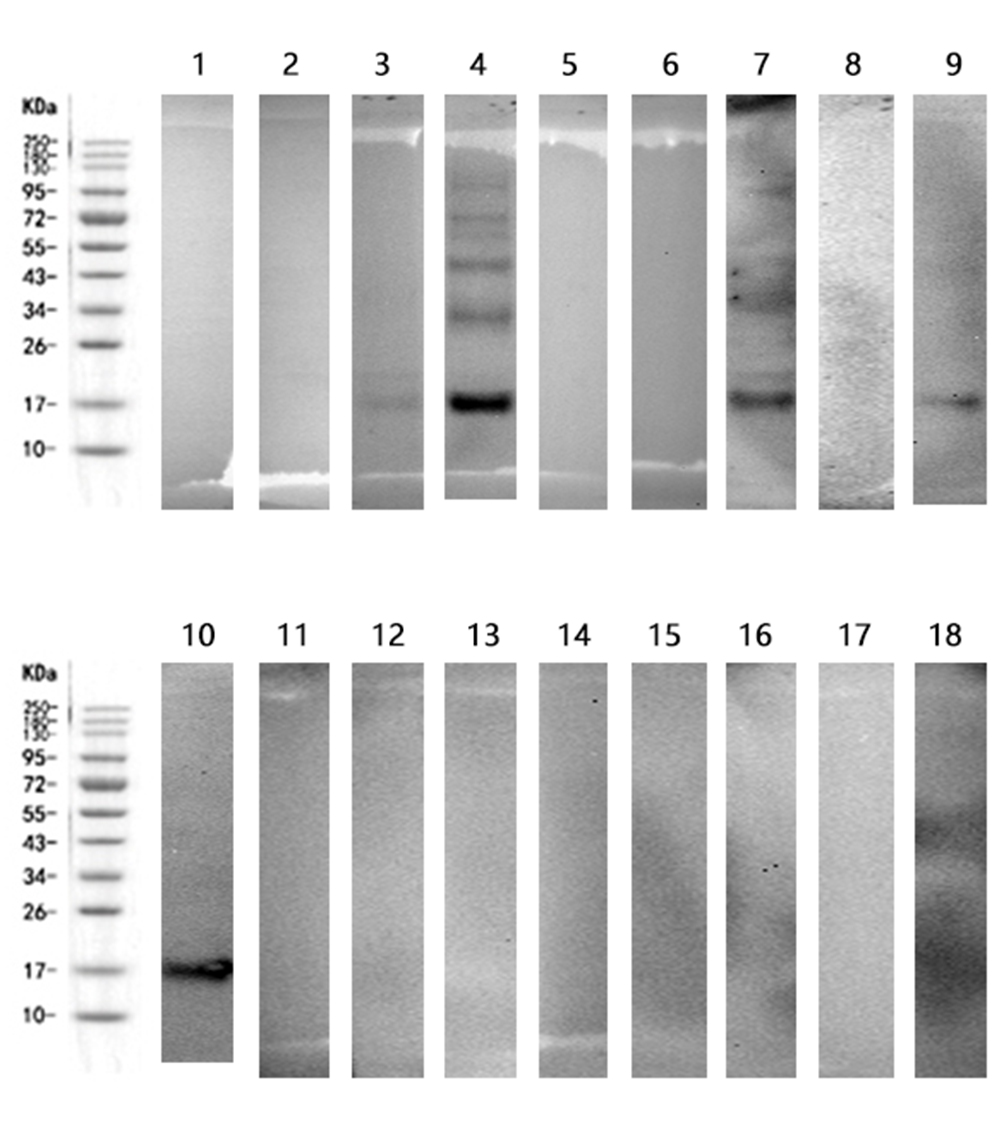


**FIGURE S6** Immunoblot probes the ArtCaM-specific IgE in other plant allergens.

**
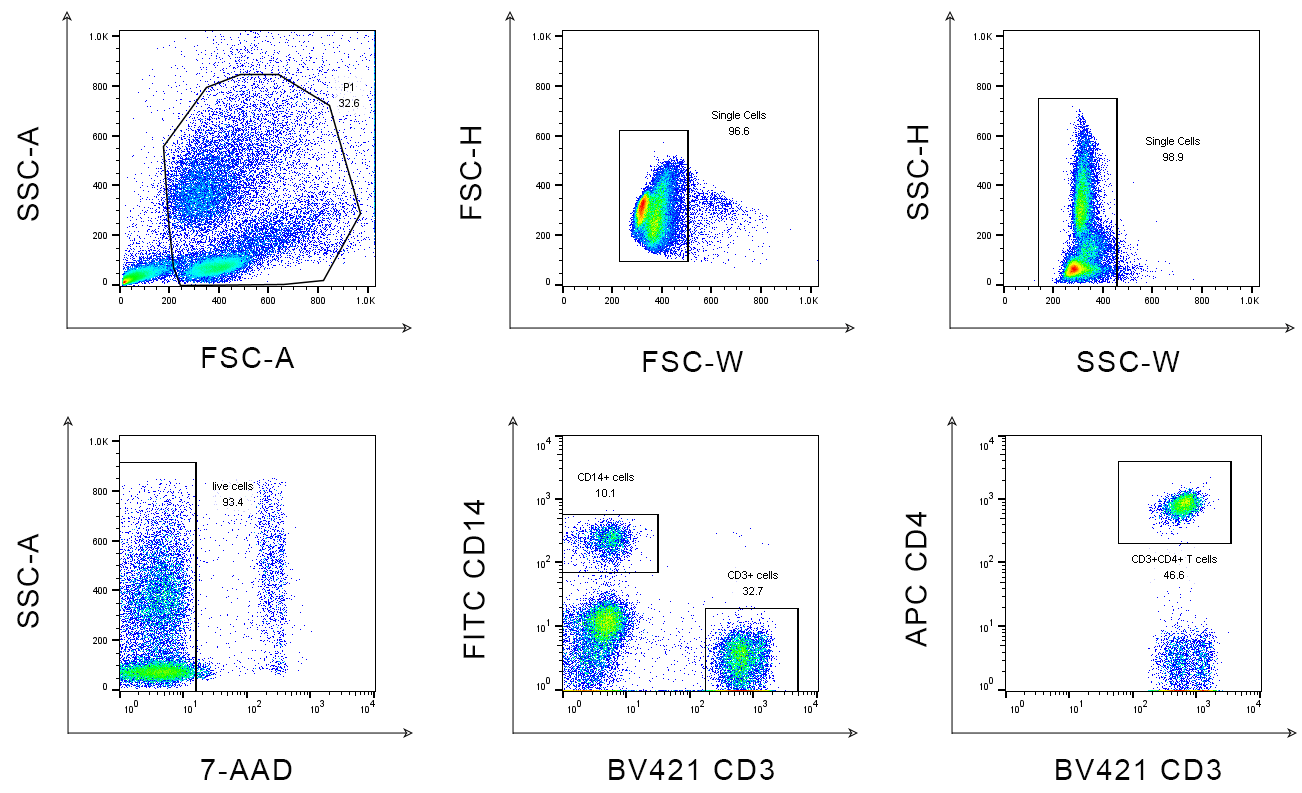
**

**FIGURE S7** The CD14+ monocytes and CD3+CD4+ T cells were sorted from PBMCs by FACS Aria II (BD Biosciences, USA).


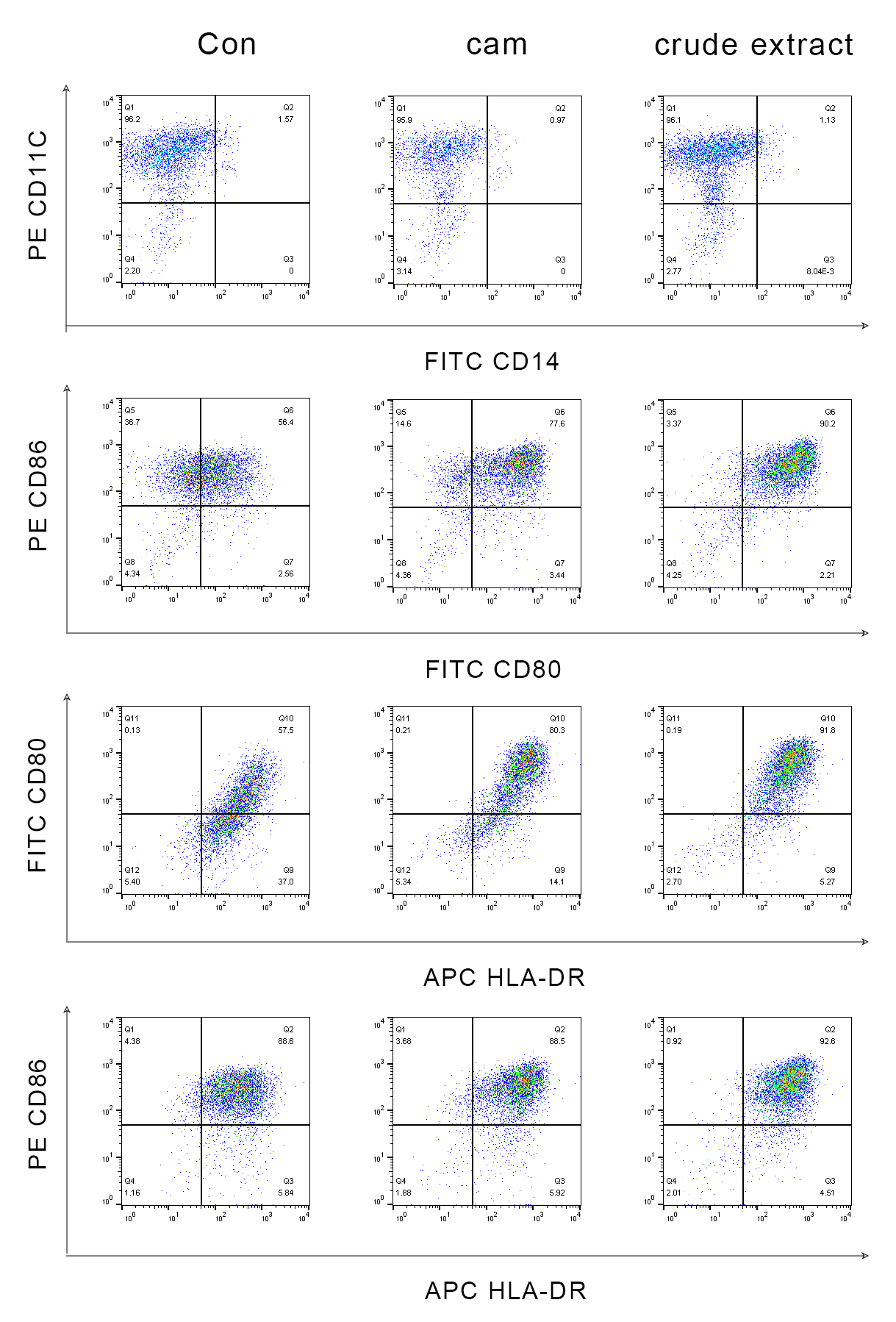


**FIGURE S8** Whole Artemisia extract had priming effect on DCs similar to ArtCaM
